# The Human Phenotype Ontology in 2021

**DOI:** 10.1093/nar/gkaa1043

**Published:** 2020-12-02

**Authors:** Sebastian Köhler, Michael Gargano, Nicolas Matentzoglu, Leigh C Carmody, David Lewis-Smith, Nicole A Vasilevsky, Daniel Danis, Ganna Balagura, Gareth Baynam, Amy M Brower, Tiffany J Callahan, Christopher G Chute, Johanna L Est, Peter D Galer, Shiva Ganesan, Matthias Griese, Matthias Haimel, Julia Pazmandi, Marc Hanauer, Nomi L Harris, Michael J Hartnett, Maximilian Hastreiter, Fabian Hauck, Yongqun He, Tim Jeske, Hugh Kearney, Gerhard Kindle, Christoph Klein, Katrin Knoflach, Roland Krause, David Lagorce, Julie A McMurry, Jillian A Miller, Monica C Munoz-Torres, Rebecca L Peters, Christina K Rapp, Ana M Rath, Shahmir A Rind, Avi Z Rosenberg, Michael M Segal, Markus G Seidel, Damian Smedley, Tomer Talmy, Yarlalu Thomas, Samuel A Wiafe, Julie Xian, Zafer Yüksel, Ingo Helbig, Christopher J Mungall, Melissa A Haendel, Peter N Robinson

**Affiliations:** Ada Health GmbH, Berlin, Germany; Monarch Initiative; Monarch Initiative; The Jackson Laboratory for Genomic Medicine, Farmington, CT, USA; Monarch Initiative; Semanticly Ltd, London, UK; European Bioinformatics Institute (EMBL-EBI); Monarch Initiative; The Jackson Laboratory for Genomic Medicine, Farmington, CT, USA; Translational and Clinical Research Institute, Newcastle University, Newcastle upon Tyne, UK; Clinical Neurosciences, Newcastle upon Tyne Hospitals NHS Foundation Trust, Newcastle upon Tyne, UK; Monarch Initiative; Oregon Clinical & Translational Research Institute, Oregon Health & Science University; Department of Neurosciences, Rehabilitation, Ophthalmology, Genetics, and Maternal and Child Health, University of Genoa, Genoa, Italy; Pediatric Neurology and Muscular Diseases Unit, IRCCS ‘G. Gaslini’ Institute, Genoa, Italy; Western Australian Register of Developmental Anomalies, King Edward memorial Hospital, Perth, Australia; Telethon Kids Institute and the Division of Paediatrics, Faculty of Helath and Medical Sciences, University of Western Australia, Perth, Australia; American College of Medical Genetics and Genomics (ACMG), Bethesda, MD, USA; Computational Bioscience Program, University of Colorado Anschutz Medical Campus, Colorado, USA; Johns Hopkins University Schools of Medicine, Public Health, and Nursing; Department of Pediatrics, Dr. von Hauner Children's Hospital, University Hospital, Ludwig-Maximilians-Universität München, Munich, Germany; Division of Neurology, Children's Hospital of Philadelphia, Philadelphia, PA, USA; Department of Biomedical and Health Informatics (DBHi), Children's Hospital of Philadelphia, Philadelphia, PA, USA; Division of Neurology, Children's Hospital of Philadelphia, Philadelphia, PA, USA; Department of Biomedical and Health Informatics (DBHi), Children's Hospital of Philadelphia, Philadelphia, PA, USA; Department of Pediatrics, Dr. von Hauner Children's Hospital, University Hospital, Ludwig-Maximilians-Universität München, Munich, Germany; Ludwig-Maximilians University, German Center for Lung Research (DZL), Munich, Germany; Ludwig Boltzmann Institute for Rare and Undiagnosed Diseases, Vienna, Austria; CeMM Research Center for Molecular Medicine of the Austrian Academy of Sciences, Vienna, Austria; Ludwig Boltzmann Institute for Rare and Undiagnosed Diseases, Vienna, Austria; CeMM Research Center for Molecular Medicine of the Austrian Academy of Sciences, Vienna, Austria; Institute for Systems Genomics, University of Connecticut, Farmington, CT 06032, USA; INSERM, US14––Orphanet, Plateforme Maladies Rares, Paris, France; Monarch Initiative; Environmental Genomics and Systems Biology, Lawrence Berkeley National Laboratory, Berkeley CA, USA; American College of Medical Genetics and Genomics (ACMG), Bethesda, MD, USA; Department of Pediatrics, Dr. von Hauner Children's Hospital, University Hospital, Ludwig-Maximilians-Universität München, Munich, Germany; Department of Pediatrics, Dr. von Hauner Children's Hospital, University Hospital, Ludwig-Maximilians-Universität München, Munich, Germany; German Centre for Infection Research (DZIF), Munich, Germany; Unit for Laboratory Animal Medicine, Department of Microbiology and Immunology, Center for Computational Medicine and Bioinformatics, University of Michigan Medical School, Ann Arbor, MI, USA; Department of Pediatrics, Dr. von Hauner Children's Hospital, University Hospital, Ludwig-Maximilians-Universität München, Munich, Germany; FutureNeuro, SFI Research Centre for Chronic and Rare Neurological Diseases, Ireland; Institute for Immunodeficiency, Center for Chronic Immunodeficiency (CCI). Faculty of Medicine, Medical Center - University of Freiburg, Freiburg, Germany; Centre for Biobanking FREEZE, Faculty of Medicine, Medical Center - University of Freiburg, Freiburg, Germany; Department of Pediatrics, Dr. von Hauner Children's Hospital, University Hospital, Ludwig-Maximilians-Universität München, Munich, Germany; Department of Pediatrics, Dr. von Hauner Children's Hospital, University Hospital, Ludwig-Maximilians-Universität München, Munich, Germany; Ludwig-Maximilians University, German Center for Lung Research (DZL), Munich, Germany; Luxembourg Centre for Systems Biomedicine, University of Luxembourg, L-4367 Belvaux, Luxembourg; INSERM, US14––Orphanet, Plateforme Maladies Rares, Paris, France; Monarch Initiative; Translational and Integrative Sciences Center, Department of Environmental and Molecular Toxicology, Oregon State University, OR, USA; American College of Medical Genetics and Genomics (ACMG), Bethesda, MD, USA; Monarch Initiative; Translational and Integrative Sciences Center, Department of Environmental and Molecular Toxicology, Oregon State University, OR, USA; American College of Medical Genetics and Genomics (ACMG), Bethesda, MD, USA; Department of Pediatrics, Dr. von Hauner Children's Hospital, University Hospital, Ludwig-Maximilians-Universität München, Munich, Germany; Ludwig-Maximilians University, German Center for Lung Research (DZL), Munich, Germany; INSERM, US14––Orphanet, Plateforme Maladies Rares, Paris, France; WA Register of Developmental Anomalies; Curtin University, Western Australia, Australia; Division of Kidney-Urologic Pathology, Johns Hopkins University, Baltimore, MD 21205, USA; SimulConsult, Inc., Chestnut Hill, MA, USA; Research Unit for Pediatric Hematology and Immunology, Division of Pediatric Hemato-Oncology, Department of Pediatrics and Adolescent Medicine, Medical University of Graz, Graz, Austria; The William Harvey Research Institute, Charterhouse Square Barts and the London School of Medicine and Dentistry Queen Mary University of London, London EC1M 6BQ, UK; Genomic Research Department, Emedgene Technologies, Tel Aviv, Israel; Faculty of Medicine, Hebrew University Hadassah Medical School, Jerusalem, Israel; West Australian Register of Developmental Anomalies, East Perth, WA, Australia; Rare Disease Ghana Initiative, Ghana; Division of Neurology, Children's Hospital of Philadelphia, Philadelphia, PA, USA; The Epilepsy NeuroGenetics Initiative (ENGIN), Children's Hospital of Philadelphia, PA, USA; Human Genetics, Bioscientia GmbH, Ingelheim, Germany; Department of Neurology, University of Pennsylvania, Perelman School of Medicine, Philadelphia, PA, USA; The Epilepsy NeuroGenetics Initiative (ENGIN), Children's Hospital of Philadelphia, Philadelphia, PA, USA; Monarch Initiative; Environmental Genomics and Systems Biology, Lawrence Berkeley National Laboratory, Berkeley CA, USA; Monarch Initiative; Oregon Clinical & Translational Research Institute, Oregon Health & Science University; Translational and Integrative Sciences Center, Department of Environmental and Molecular Toxicology, Oregon State University, OR, USA; Monarch Initiative; The Jackson Laboratory for Genomic Medicine, Farmington, CT, USA; Institute for Systems Genomics, University of Connecticut, Farmington, CT 06032, USA

## Abstract

The Human Phenotype Ontology (HPO, https://hpo.jax.org) was launched in 2008 to provide a comprehensive logical standard to describe and computationally analyze phenotypic abnormalities found in human disease. The HPO is now a worldwide standard for phenotype exchange. The HPO has grown steadily since its inception due to considerable contributions from clinical experts and researchers from a diverse range of disciplines. Here, we present recent major extensions of the HPO for neurology, nephrology, immunology, pulmonology, newborn screening, and other areas. For example, the seizure subontology now reflects the International League Against Epilepsy (ILAE) guidelines and these enhancements have already shown clinical validity. We present new efforts to harmonize computational definitions of phenotypic abnormalities across the HPO and multiple phenotype ontologies used for animal models of disease. These efforts will benefit software such as Exomiser by improving the accuracy and scope of cross-species phenotype matching. The computational modeling strategy used by the HPO to define disease entities and phenotypic features and distinguish between them is explained in detail.We also report on recent efforts to translate the HPO into indigenous languages. Finally, we summarize recent advances in the use of HPO in electronic health record systems.

## INTRODUCTION

The Human Phenotype Ontology (HPO) is a comprehensive resource that systematically defines and logically organizes human phenotypes. As an ontology, HPO enables computational inference and sophisticated algorithms that support combined genomic and phenotypic analyses. Broad clinical, translational and research applications using the HPO include genomic interpretation for diagnostics, gene-disease discovery, mechanism discovery and cohort analytics, all of which assist in realizing precision medicine. We have developed open community resources consisting of the HPO ontology and a comprehensive corpus of disease HPO phenotype annotations (HPOA) corresponding to each of nearly eight thousand rare diseases. Together with other terminologies and classifications, the HPO and its disease annotations enable semantic interoperability in digital medicine. Community contributions have added depth, coverage, and sophistication to the HPO since its founding in 2008 (1–4). The HPO team welcomes additional contributions from consortia or individuals; see https://hpo.jax.org/app/help/collaboration.

The HPO differs from other available clinical terminologies in several crucial ways. First, the HPO has substantially deeper and broader coverage of phenotypes than any other clinical terminology. In 2014, Bodenreider and colleagues compared the HPO’s coverage of phenotypes to the combined coverage of all other relevant terminologies in the United Medical Language System (UMLS) and found that the UMLS resources covered only about 35% of the concepts in the HPO (5). This led to the HPO being incorporated into the UMLS (in collaboration with the HPO team). Second, the HPO is not a simple terminology, but rather a full Web Ontology Language (OWL) ontology and thus a computational resource that allows sophisticated analyses, including logical inference (6). Finally, the HPO-based computational disease models are utilized within most, if not all, current phenotype-driven genomic diagnostics software (7–15).

**Figure 1.**
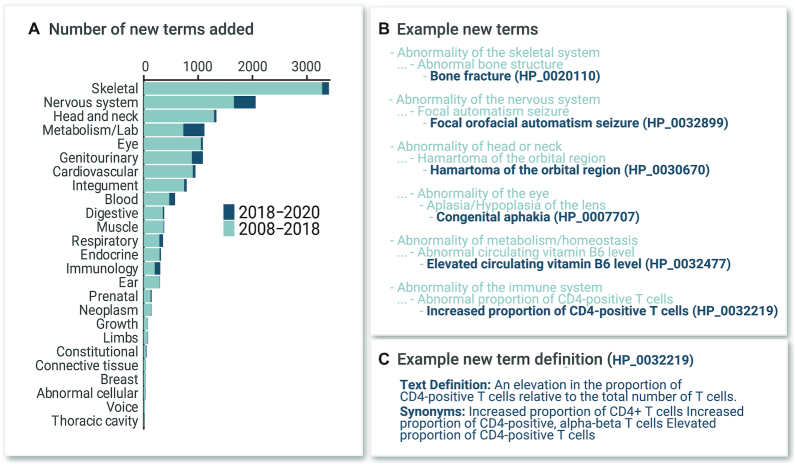
HPO terms organized by organ system. (**A**) Counts for top-level phenotype terms (direct descendants of Phenotypic abnormality (HP:0000118) are shown. Counts of terms added to the ontology after the previous article in this series (19) are shown in dark blue (added between 25 July 2018 and 18 August 2020). (**B**) Examples of new terms added 2018–2020 and their parent terms, for selected organ systems. (**C**) An example text definition and synonyms for a new term.

As of 15 September 2020, the HPO contained 15 247 terms, representing a 9.3% increase since the last Nucleic Acids Research (NAR) manuscript (Figure 1). The HPOAs are computational disease models with associated HPO terms. For instance, the disease Marfan syndrome is characterized by—and therefore annotated to—over 50 phenotypic abnormalities including Aortic aneurysm (HP:0004942) (each abnormality is represented by an HPO term). The annotations can have modifiers that describe the age of onset and the frequencies of features. For instance, the phenotypic abnormality Brachydactyly (HP:0001156) is rare in Hydrolethalus syndrome (3/56 according to a published study referenced in our data) but affects nearly 100% of patients diagnosed with most of the 484 other diseases annotated to this term. This type of information can be used by algorithms to weight findings in the context of clinical differential diagnosis (16).

The HPO provides annotations to diseases defined by Online Mendelian Inheritance in Man (OMIM) (17), nearly all of which are monogenic (Mendelian) diseases. Currently, 93 885 of a total of 108 580 such annotations were derived from mining the Clinical Synopsis section of the corresponding entry. 14 695 (13.5%) annotations were produced by curation by the HPO team and often contain additional information such as age of onset, affected sex, clinical modifiers, or overall frequency of the feature. A total of 7801 diseases are annotated in this way, corresponding to 108 580 annotations in all (with a mean of 13.9 annotations per disease). 296 curated annotations to 47 chromosomal diseases identified by DECIPHER (18) accessions were also generated by the HPO team (mean 6.2 annotations per disease).

**Figure 2.**
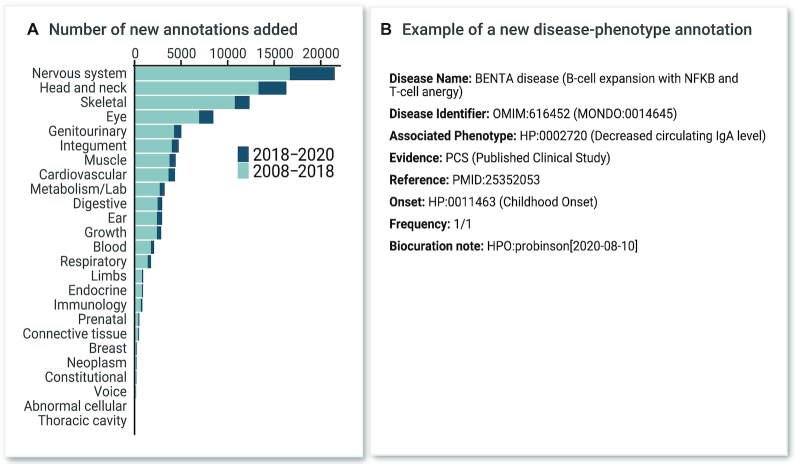
Annotations. Disease annotations using HPO terms organized by organ system. (**A**) Annotation counts for top-level phenotype terms (direct descendants of Phenotypic abnormality HP:0000118) are shown. Counts of annotations added to the ontology after the previous article in this series (19) are shown in dark blue (added between 25 July 2018 and 18 August 2020). Short forms are used to indicate the top level terms; for instance, ‘Ear’ indicates Abnormality of the ear (HP:0000598). (**B**) Example new annotation.

In parallel, Orphanet uses the HPO to annotate rare diseases and has continued to develop annotations to a broad range of diseases (currently 96 612 annotations utilizing 7495 distinct HPO terms for 3956 diseases, with an average of 24.4 terms per disease). These annotations include information about the frequency (obligatory, very frequent, frequent, occasional, very rare or excluded) and whether the annotated HPO term is a major diagnostic criterion or a pathognomonic sign of the rare disease. These data are available at Orphadata.org and in the HPO-Orphanet Rare Disease Ontology (ORDO) ontological module called HOOM (See Data Availability section, below). While some of the annotated diseases overlap, Orphanet contains information about non-Mendelian rare diseases and defines diseases primarily based on clinical criteria, thereby providing a complementary resource. Both sets of annotations are available in a combined annotation file available on the HPO website. Figure 2 displays the growth in annotations to the OMIM entries.

Abnormal phenotypic features or manifestations of human disease stored in HPO are also employed for medical research projects such as SOLVE-RD. Funded by the European Commission, SOLVE-RD aims to solve large numbers of rare diseases for which a molecular cause is not known.

The HPO has a sophisticated quality control pipeline. In addition to custom software, we make extensive use of the quality control checks implemented in ROBOT (‘ROBOT is an OBO Tool’) (47). We have added descriptions of our quality control processes to the HPO website under the Help menu.

## COMMUNITY COLLABORATIONS TO EXTEND THE COVERAGE OF HPO

The UK’s National Institute for Health Research (NIHR) Rare Disease initiatives extensively use the HPO in their RD-TRC (Rare Disease––Translational Research Collaboration) and NIHR BioResource, in wide-ranging studies. Following an HPO workshop with members of the NIHR-RD-TRC in 2017, the NIHR-RD-TRC assessed the maturity of the HPO across different disease areas and organ systems. Disorders of the immune system, central nervous system, the respiratory system, and the kidney were among the areas where additional work was deemed desirable (3). In this article, we report on our work in these areas with clinical experts.

### Epilepsy

**Figure 3.**
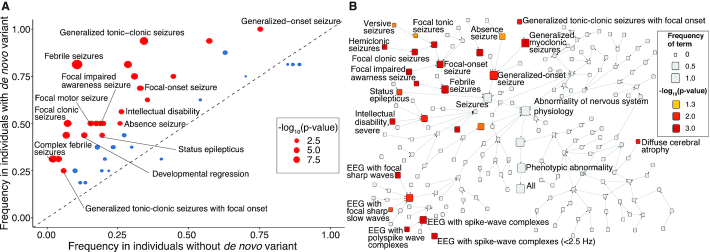
HPO-based analyses demonstrate the clinical features associated with diagnostic variants in *SCN1A* in published cohorts with developmental and epileptic encephalopathies of various known, or unknown but presumed genetic, etiologies. Fisher's exact test p-value for each term indicates the significance of the association between the HPO term and the presence of a diagnostic *SCN1A* variant in the cohort. (**A**) The frequency of HPO terms in *SCN1A* variant carriers versus non-carriers regardless of age. (**B**) The same data presented to demonstrate the conceptual relationships between associated features within the structure of the HPO. (A) and (B) modified from (24) with only a selection of terms labeled for legibility.

The epilepsies are a group of diverse disorders that share a predisposition to seizures (20). They are phenotypically complex with constellations of clinical features indicating different age-specific syndromes, broad epilepsy types, and etiologies that guide clinical management (21). We have recently demonstrated that phenotypic similarity approaches based on HPO-related phenotypes in the epilepsies can be used to identify novel genetic etiologies such as *AP2M1* (22), to map the natural history of genetic epilepsies over time from electronic medical records (23), and to identify patterns of gene-phenotype associations (Figure 3) (24).

Given the release of a new International League Against Epilepsy (ILAE) seizure classification (25), a revision of the seizure subontology of the HPO was performed, supported by the ILAE Epilepsiome Task Force. This project commenced with a week-long workshop in 2018 followed by fortnightly teleconferences held over the following year to coordinate a draft ontology created on WebProtégé (26). In addition to the new classification of seizure types (25), the new subontology integrates concepts from other proposed classifications of status epilepticus (27), reflex seizures (28), neonatal seizures (29), seizure semiology (30) and the literature of febrile seizures (31–34).

**Figure 4.**
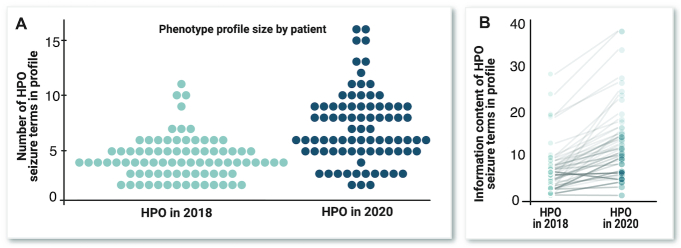
(**A**) The number of seizure terms applicable to the same clinical data from 82 individuals, and (**B**) the total information content of seizure terms of the same individuals according to the new and previous HPO seizure subontologies, where the information content of each term is equal to the negative logarithm of the proportion of individuals annotated with the term (Lewis-Smith *et al.*, manuscript in preparation).

An important challenge in seizure classification is that seizures are paroxysmal, and often incompletely characterized or observed. In order to maximize the available information, the revised subontology includes terms independent of some of the dimensions of seizure description. For example, the terms Focal aware seizure (HP:0002349) and Focal motor seizure (HP:0011153) allow a true instance of Focal aware motor seizure (HP:0020217) to be coded as precisely as possible when knowledge of either the initial manifestation or the preservation of awareness is unknown. These concepts provide a way to categorize high-level, incomplete information that often makes disease classification difficult. Where possible, pre-existing terms were retained for the benefit of legacy HPO data. A few inconsistencies with contemporary seizure concepts were identified and corrected, such as the previous relationship of Bilateral tonic-clonic seizure with focal onset (HP:0007334) as a type of Generalized-onset seizure (HP:0002197) rather than Focal-onset seizure (HP:0007359). The new seizure subontology currently contains 347 terms, which significantly increases the detail with which seizures can be described (Figure 4).

### Inborn errors of immunity (IEI)

Inborn errors of immunity (IEI), previously referred to as primary immunodeficiencies (PID), involve a variable, disorder-specific predisposition towards infections, immune dysregulation (including autoimmunity, autoinflammation, granuloma formation, lymphoproliferation, etc.), and malignancies. Phenotypes of IEI are often complex, making it difficult to distinguish primary disease-specific features from secondary unspecific, infection- or inflammation-related, or merely randomly occurring clinical manifestations. However, unequivocal phenotypic descriptions are needed for semantic interoperability to enable the use of defining, cross-referencing, and/or filtering algorithms during the process of diagnosing these rare diseases. For the purpose of data verification of entries into the large international registry of the European Society for Immunodeficiencies (ESID) that includes data from >30 000 patients, either a known genetic diagnosis or the fulfillment of working definitions for the clinical diagnosis of IEI is required. Together with a group of international collaborators, the ESID registry working group designed a comprehensive list of obligatory and optional criteria for 92 entities that lack a genetic diagnosis (e.g. common variable immunodeficiency) that were cross-validated by other experts in a two-phase process (35). To enhance this catalog of clinical working definitions of IEI, we recently added HPO terms and the frequencies of phenotypes observed, derived from HOOM. For most other IEIs that are included in the genotypic classification of the International Union of Immunological Societies (36), complete HPO term annotations are still lacking. To improve the available vocabulary and annotated diseases, a targeted expansion of IEI relevant HPO terms and re-annotation of currently known IEIs was launched by representatives of the ESID genetics working party and of ERN-RITA (European Network on Rare Primary Immunodeficiency, Autoinflammatory and Autoimmune diseases) with input from the International Society of Systemic Autoinflammatory Diseases (ISSAID) in 2018. The systematic review involved expert clinicians, geneticists, researchers (working on IEI) and bioinformaticians combining an ontology-guided machine-learning approach (37) with expert clinical immunologists’ reviews (M. Haimel, *et al.*, manuscript in preparation). The HPO-classification of IEI is part of The Medical Informatics Initiative Germany (MII) founded by the Federal Ministry of Education and Research, which has launched the Collaboration on Rare Diseases (CORD) project. Aided by the national TRANSLATE-NAMSE project, this initiative plays a key role in the development of digitalized patient data allowing clinicians and scientists to make use of standardized phenotypic patient information. Digital recording of HPO terms will facilitate genetic research to identify disease-causing variants; it will also support large-scale studies aiming to associate genetic variance with a plethora of risks that can disrupt immune homeostasis.

### Kidney Precision Medicine Project (KPMP)

**Figure 5.**
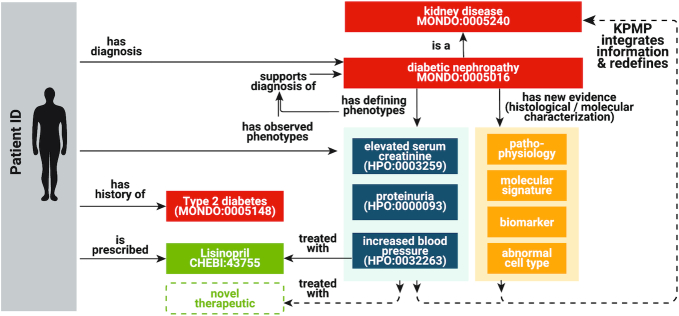
One major goal of KPMP is to refine classification of kidney diseases in molecular, cellular, and phenotypic terms and thereby identify novel targeted therapies. The kidney-related HPO terms are being used in multiple ways in KPMP. For example, KPMP has used the HPO terms for clinical and pathological phenotype annotations, integrative Kidney Tissue Atlas Ontology (KTAO) (39) development, and systematic data integration software development.

The Kidney Precision Medicine Project (KPMP) aims to understand and find ways to treat chronic kidney disease (CKD) and acute kidney injury (AKI). KPMP has contributed over 100 kidney-related phenotype terms; clinical nephrologists, pathologists and ontologists worked together over multiple workshops to propose new terms and modifications to HPO and underlying ontologies such as Uberon (38). Two new major HPO branches were generated, one focusing on pathology-related terms, and the other on clinical phenotype terms (Figure 5).

### Pulmonology

The category of respiratory disorders is not only underrepresented in the HPO; it is rapidly expanding with the ongoing molecular definition of rare to ultra-rare novel diseases. Therefore, substantial effort was undertaken to improve the foundation and formulation of terms and disease associations. However, gaps remain–for example, for most rare and common pulmonary disorders included in the current classification of children's interstitial lung diseases (40), comprehensive HPO term annotations still need to be completed. To this end, representatives of the European research collaboration for Children's Interstitial Lung Disease (chILD-EU) consortium have called for community participation and initiated a low barrier approach to facilitate contribution to the HPO for newcomers (see section on contributing to the HPO in the Data Availability section, below). To facilitate sharing knowledge about rare respiratory disorders, information is collected in international registers like the Kids Lung Register, operating through the chILD-EU management platform. The chILD-EU network utilizes the HPO, which significantly improved the categorization of novel diseases and the annotation of cases included for long term investigation (41).

### Pharmacogenomics

HPO has introduced several terms to describe drug response phenotypes. The new terms added to HPO are branched under the term Abnormal drug response (HP:0020169) and aim to encompass a spectrum of clinical phenotypes with regards to drug metabolism. The underlying HPO terms refer to abnormal blood concentration of drugs, altered efficacy and adverse drug response. As pharmacogenomic research makes its way into routine clinical applications, such terms may be valuable in describing variance in drug metabolism as ascertained by laboratory investigation or genetic sequencing (42).

### Newborn screening

Screening of newborns to facilitate the early identification, diagnosis and treatment of rare diseases occurs throughout the world. In the United States, the Newborn Screening Translational Research Network (NBSTRN) provides tools and resources to researchers working to discover novel screening technologies and interventions (43). An important goal for the NBSTRN is to understand health outcomes and the natural history of rare diseases by capturing longitudinal genomic and phenotypic information on the estimated 22 000 infants diagnosed through newborn screening (NBS) each year. A US federal advisory committee recommends conditions for NBS resulting in the Recommended Uniform Screening Panel, and in 2018, screening for Spinal Muscular Atrophy was endorsed. As a case study of HPO in NBS and rare disease, a REDCap™ data dictionary of 4757 data elements in the SPOT SMA Longitudinal Pediatric Data Resource was reviewed to identify existing terms and suggest new terms. The aim of this effort is to develop HPO as a resource for the longitudinal followup of NBS identified individuals with the goal of advancing understanding of rare disease.

### Interoperability with other phenotype ontologies

We have developed templated ontology design patterns to structure OWL definitions, encoded as Dead Simple OWL Design Patterns (DOSDPs) (44). DOSPDs provide a number of advantages, including standardized patterns for the logical definitions and automatic classification. As coordinators of the Phenotype Ontologies Reconciliation Effort (45, 46), HPO developers contributed to the definition of 207 DOSDP templates for the consistent definition of phenotypes across species and modalities (44). The Unified Phenotype Ontology (uPheno) integrates multiple phenotype ontologies into a harmonized cross-species phenotype ontology. uPheno enables the comparison and grouping of species-specific phenotypes under species-neutral categories, and links phenotypes from one species with comparable phenotypes from other species. Using templates generates phenotype terms that are not only consistently structured, but also enriched with associations to, for example, biological processes (Gene Ontology), anatomical entities, and molecular entities. For example, an abnormal level of chemical entity with role in location provides a template for terms such as Abnormal circulating hormone level (HP:0003117). Reconciliation is ongoing and is improving the alignment between phenotype ontologies for a range of organisms including *C. elegans*, *Dictyostelium discoideum*, *Drosophila*, fission yeast, planarian, *Xenopus*, mammals (MP) and zebrafish (ZP), as well ontologies for glycophenotypes (47) and pathogen–host interactions. The goal is to enable meaningful and reliable mapping of phenotype data such as gene-to-phenotype associations across databases that are specific to particular modalities or organisms, and leverage this data for a variety of important applications including clinical diagnosis and variant prioritization. For example, Exomiser (15) leverages the semantic associations between HPO, MP and ZP to prioritize variants effectively by matching human phenotypic abnormalities with phenotypes observed in animal models with knockouts of genes orthologous to human disease-associated genes.

**Figure 6.**
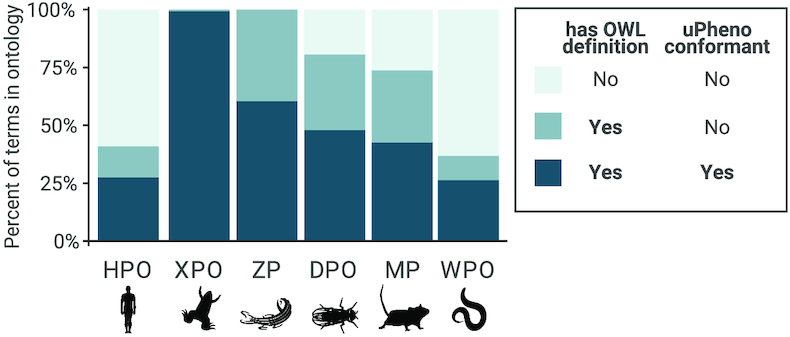
Proportions of terms defined in the HPO, the Mammalian Phenotype Ontology (MP) (48), the Drosophila Phenotype Ontology (DPO) (49), the Worm Phenotype Ontology (WPO) (50), the Xenopus Phenotype Ontology (XPO) (51) and the Zebrafish Phenotype Ontology (ZP) (52).

Figure 6 illustrates the extent to which phenotype ontologies adhere to phenotype DOSDP patterns (‘uPheno conformant’). Currently, the HPO has 6154 OWL-defined terms (41% of the total number of 15 029 terms), out of which 4139 (67%) adhere to an existing template. While some phenotypes may be too complex to define using a general template, we hope to increase our coverage to ∼50% of the terms.

### Indigenous languages

For equity and scale of precision medicine and precision public health, it is critical to advance methods to improve the diagnosis and treatment of rare diseases. Communication is critical to healthcare and methods to deliver and incorporate translations, community narratives and family-based approaches are important to advancing culturally appropriate care. Lyfe Languages (lyfelanguages.com) is improving communication between indigenous patients, families, and medical professionals, in part by delivering indigenous language translations of the HPO. This started with a focus on rare diseases, then expanded to also include COVID-19 and is being extended into mental health. Currently, HPO terms are being translated to 11 Australian Aboriginal and Torres Strait Islander Languages and 6 Ghanian indigenous languages. The latter project is being performed together with the Rare Disease Ghana Initiative.

### HPO for medical education & crowdsourcing

One of the advantages of the structured knowledge contained in the HPO is that it can be utilized as a teaching tool. One recent example of using HPO in this way is Phenotate, a portal that allows the annotation of OMIM and Orphanet disorders with HPO terms to be formulated as assignments for students (53). Phenotate has been used in five undergraduate courses, allowing for the collection of annotations for 22 diseases, including six where previously structured annotations were not available. Interestingly, the annotations generated by Phenotate, while sourced from untrained undergraduate students, were equal to curated gold standards in terms of allowing clinicians to identify rare disorders.

## EHR INTEGRATION

**Figure 7.**
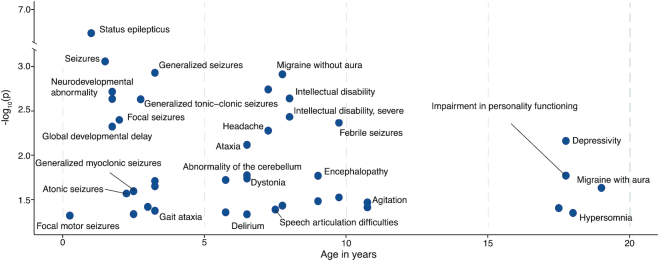
Analysis of time-stamped EHRs of children with epilepsy demonstrates the association of HPO terms with diagnostic *SCN1A* variants at different ages (modified from (23) with only a selection of terms labeled for legibility).

Electronic health records (EHRs) have been widely adopted and offer an unprecedented opportunity to accelerate translational research because of advantages of scale and cost-efficiency as compared to traditional cohort-based studies. Textual data within EHRs can describe phenotypic features that are not encoded within the structured fields of the EHR, but natural language processing (NLP) is required to transform such data into terminological entities (ontology terms) for downstream analysis. NLP of phenotypic data is becoming a mature field that can be used to improve clinical care, and HPO has been used by a number of groups as a resource for EHR analysis (54). For example, EHRs spanning individuals’ entire childhoods can be mapped to the HPO, yielding longitudinal patterns of phenotypic features associated with particular genetic etiologies (Figure 7) (23). However, EHR data are often incomplete or incorrect, and EHR systems are generally billing instruments rather than tools to improve patient care, much less allow secondary research.

LOINC (Logical Observations Identifiers, Names, Codes) is a clinical terminology for laboratory test orders and results that is widely used in EHRs (55). We developed a mapping strategy (LOINC2HPO) to transform laboratory data in EHR records to HPO terms. For instance, if the result of the test LOINC:6298-4 (*potassium in blood*) is above normal limits, our library would call the HPO term Hyperkalemia (HP:0002153). Many common tests in medicine can be performed in multiple ways, so there can be multiple LOINC codes for tests that measure the same biological quantity. For instance, currently, there are four different LOINC terms for different tests of urine nitrite. Our library maps these terms to the same HPO term. Additionally, the hierarchy of the HPO can be used to roll up related results (e.g. reduced concentrations of different B vitamins in the blood). In a pilot study, we investigated EHR data from 15 681 patients with respiratory complaints and identified known biomarkers for asthma (56). However, the absence of an ontological structure in LOINC, a known issue, impeded optimal information capture and coding. Members contributing to last year's paper have secured funding to partner with the LOINC developer to address this challenge, which will enhance the community's ability to categorize clinical laboratory findings into HPO terms.

The diagnostic decision support system SimulConsult uses a controlled list of 9871 findings chosen for their importance in diagnosis (12). As part of a project to use machine-assisted chart review to flag which of those findings are discussed in the EHR, hundreds of new findings were added to HPO in a collaboration between HPO and SimulConsult. Since HPO is one of the key inputs to the UMLS concept codes, adding terms to HPO is an efficient workflow for adding terms to UMLS as well.

Enabling large scale integration of biomedical knowledge with clinical patient data requires robust and accurate mappings between standardized clinical terminology concepts and ontologies, like the HPO. Existing work has demonstrated the power of the HPO to enrich clinical data including craniofacial and oral phenotypes (57), rare and Mendelian disease (58, 59), and infectious disease (60). There have also been more generalized mapping efforts aimed at aligning different clinical terminologies to the HPO including free-text narratives (61) and structured data like diagnosis codes (62, 63). While this work is very promising, it has largely been limited to specific clinical domains (i.e. only diagnosis codes from structured data or only phenotype mentions in free-text). Additionally, the vast majority of prior work focused on mapping clinical codes from standardized terminologies has exclusively focused on mapping only specific terminologies (e.g. SNOMED-CT or ICD-9). Mapping to a single terminology limits the generalizability of the mappings. One solution is to generate mappings to common data models (CDM) as well as tools that integrate different EHR data, such as Informatics for Integrating Biology and the Bedside (i2b2) (64) and Observational Health Data Sciences and Informatics's Observational Medical Outcomes Partnership (OMOP) (65).

Currently, there exist no large-scale mappings spanning multiple clinical domains (e.g. diagnosis, medications, laboratory measurements) to the HPO and other biomedical ontologies. In collaboration with researchers from the University of Colorado Anschutz Medical Campus, a new framework, OMOP2OBO (66), is being developed to map several ontologies, including the HPO, to standardized clinical terminologies in the OMOP CDM. The mappings are generated using a combination of manual and automatic approaches and validated by a panel of clinical and biological domain experts. To date, the mappings cover over 29 000 diagnosis codes (over 20 000 diagnosis codes map to a total of over 4000 HPO codes), 1700 medication ingredients, and over 11 000 laboratory test results including and extending current LOINC2HPO annotations.

### The distinction between diseases and phenotypes

The community uses the word *phenotype* with multiple meanings. The HPO defines a disease as an entity that has all four of the following attributes:

- an etiology (whether identified or as yet unknown)
- a time course
- a set of phenotypic features
- if treatments exist, there is a characteristic response to them

A phenotype *phenotypic feature* is a part of a disease. The phenotype of an individual with a disease can be said to be the sum of all of the phenotypic features manifestated by that individual. HPO terms can be used to describe the phenotypic features that occur in individuals with a disease. For instance, if the disease entity is the common cold, then the cause is a virus; the phenotypic features include fever, cough, runny nose, and fatigue; the time course usually is a relatively acute onset with manifestations dragging on for days to about a week; and the treatment may include bed rest, aspirin, or nasal sprays. In contrast, a phenotypic feature such as fever is a manifestation of many diseases. There is a grey zone between diseases and phenotypic features. For instance, diabetes mellitus can be conceptualized as a disease, but it is also a feature of other diseases such as Bardet Biedl syndrome. The HPO takes a practical stance and provides terms for such entities. In the future, the HPO will develop tighter integration with the Mondo Disease Ontology (67) in order to define this category of HPO terms based on the corresponding diseases. A related issue is the fact that phenotypic features are analyzed and reported at different levels of granularity. For instance, the evaluation of a liver biopsy in an individual with hepatitis C would usually involve an assessment of focal lobular necrosis, portal inflammation, piecemeal necrosis, and bridging necrosis, each of which could be classified into one of several levels, each of which would be specified in the pathology report. If the findings are sufficiently abnormal, the pathologist may make a diagnosis such as chronic hepatitis. For the purposes of precision medicine, it would be preferable to have all the information available in electronic form, but in many settings, not all of this information is available. The HPO takes a practical stance, providing terms at different levels of granularity; for example, Hepatic bridging fibrosis (HP:0012852) and Chronic hepatitis (HP:0200123).

## CONCLUSION

The HPO has continued to benefit from the support of domain experts from multiple areas of clinical medicine. We will expand our work on extending the HPO terminology to several additional subontologies including those for behavioral abnormalities, various areas related to prenatal and perinatal medicine, as well as to common diseases. We are designing an online collaboration portal for domain experts to submit new disease annotations.

## DATA AVAILABILITY

Human Phenotype Ontology: https://hpo.jax.org/: Files available for download include the main ontology file in OBO, OWL, and JSON formats (See Download|Ontology); the main HPOA file, genes_to_phenotype.txt and phenotype_to_genes.txt (See Download|Annotation).

- GitHub: https://github.com/obophenotype/human-phenotype-ontology

- Change logs: https://github.com/obophenotype/human-phenotype-ontology/tree/master/src/ontology/reports

- Instructions for contributing to the HPO are available at https://hpo.jax.org/app/help/collaboration

- chILD-EU management platform: (www.childeu.net)

- Collaboration on Rare Diseases (CORD): https://www.medizininformatik-initiative.de/en/CORD

- DOSDP: https://github.com/obophenotype/upheno/tree/master/src/patterns/dosdp-dev

- ESID registry https://esid.org/Working-Parties/Registry-Working-Party/Diagnosis-criteria

- Kidney Precision Medicine Project (KPMP) https://kpmp.org/

- Lyfe languages: http://www.lyfelanguages.com/About.html

- The Medical Informatics Initiative Germany (MII): https://www.medizininformatik-initiative.de/en/start

- Monarch Initiative: https://monarchinitiative.org/

- Newborn Screening Translational Research Network (NBSTRN): www.nbstrn.org

- NIH CDE Repository: https://cde.nlm.nih.gov/.

- OMOP2OBO: https://github.com/callahantiff/OMOP2OBO

- Online Mendelian Inheritance in Man: https://omim.org/

- Orphadata (including HOOM): http://www.orphadata.org.

- Orphanet: http://www.orpha.net

- ORPHApackets: https://github.com/Orphanet/orphapacket.

- Rare Disease Ghana Initiative (https://www.rarediseaseghana.org/)

- Zooma: https://www.ebi.ac.uk/spot/zooma/

## References

[1] Robinson P.N., Köhler S., Bauer S., Seelow D., Horn D., Mundlos S. The Human Phenotype Ontology: a tool for annotating and analyzing human hereditary disease. Am. J. Hum. Genet. 2008; 83:610–615. 18950739 10.1016/j.ajhg.2008.09.017 PMC2668030

[2] Köhler S., Doelken S.C., Mungall C.J., Bauer S., Firth H.V., Bailleul-Forestier I., Black G.C.M., Brown D.L., Brudno M., Campbell J. et al. The Human Phenotype Ontology project: linking molecular biology and disease through phenotype data. Nucleic Acids Res. 2014; 42:D966–D974. 24217912 10.1093/nar/gkt1026 PMC3965098

[3] Köhler S., Vasilevsky N.A., Engelstad M., Foster E., McMurry J., Aymé S., Baynam G., Bello S.M., Boerkoel C.F., Boycott K.M. et al. The Human Phenotype Ontology in 2017. Nucleic Acids Res. 2017; 45:D865–D876. 27899602 10.1093/nar/gkw1039 PMC5210535

[4] Köhler S., Carmody L., Vasilevsky N., Jacobsen J.O.B., Danis D., Gourdine J.-P., Gargano M., Harris N.L., Matentzoglu N., McMurry J.A. et al. Expansion of the Human Phenotype Ontology (HPO) knowledge base and resources. Nucleic Acids Res. 2018; 47:D1018–D1027. 10.1093/nar/gky1105 PMC6324074 30476213

[5] Rainer W., Bodenreider O. Coverage of phenotypes in standard terminologies. Proceedings of the Joint BioOntologies and BioLINK ISMB’2014 SIG session ‘Phenotype Day.’. 2014; 41–44.

[6] Haendel M.A., Chute C.G., Robinson P.N. Classification, ontology, and precision medicine. N. Engl. J. Med. 2018; 379:1452–1462. 30304648 10.1056/NEJMra1615014 PMC6503847

[7] Sifrim A., Popovic D., Tranchevent L.-C., Ardeshirdavani A., Sakai R., Konings P., Vermeesch J.R., Aerts J., De Moor B., Moreau Y. eXtasy: variant prioritization by genomic data fusion. Nat. Methods. 2013; 10:1083–1084. 24076761 10.1038/nmeth.2656

[8] Javed A., Agrawal S., Ng P.C. Phen-Gen: combining phenotype and genotype to analyze rare disorders. Nat. Methods. 2014; 11:935–937. 25086502 10.1038/nmeth.3046

[9] Singleton M.V., Guthery S.L., Voelkerding K.V., Chen K., Kennedy B., Margraf R.L., Durtschi J., Eilbeck K., Reese M.G., Jorde L.B. et al. Phevor combines multiple biomedical ontologies for accurate identification of disease-causing alleles in single individuals and small nuclear families. Am. J. Hum. Genet. 2014; 94:599–610. 24702956 10.1016/j.ajhg.2014.03.010 PMC3980410

[10] Gurovich Y., Hanani Y., Bar O., Nadav G., Fleischer N., Gelbman D., Basel-Salmon L., Krawitz P.M., Kamphausen S.B., Zenker M. et al. Identifying facial phenotypes of genetic disorders using deep learning. Nat. Med. 2019; 25:60–64. 30617323 10.1038/s41591-018-0279-0

[11] Buske O.J., Girdea M., Dumitriu S., Gallinger B., Hartley T., Trang H., Misyura A., Friedman T., Beaulieu C., Bone W.P. et al. PhenomeCentral: a portal for phenotypic and genotypic matchmaking of patients with rare genetic diseases. Hum. Mutat. 2015; 36:931–940. 26251998 10.1002/humu.22851 PMC5467641

[12] Fuller G. Simulconsult: www.simulconsult.com. J. Neurol. Neurosurg. Psychiatry. 2005; 76:1439–1439.

[13] Firth H.V., Richards S.M., Bevan A.P., Clayton S., Corpas M., Rajan D., Van Vooren S., Moreau Y., Pettett R.M., Carter N.P. DECIPHER: database of chromosomal imbalance and phenotype in humans using ensembl resources. Am. J. Hum. Genet. 2009; 84:524–533. 19344873 10.1016/j.ajhg.2009.03.010 PMC2667985

[14] Pontikos N., Yu J., Moghul I., Withington L., Blanco-Kelly F., Vulliamy T., Wong T.L.E., Murphy C., Cipriani V., Fiorentino A. et al. Phenopolis: an open platform for harmonization and analysis of genetic and phenotypic data. Bioinformatics. 2017; 33:2421–2423. 28334266 10.1093/bioinformatics/btx147

[15] Smedley D., Jacobsen J.O.B., Jäger M., Köhler S., Holtgrewe M., Schubach M., Siragusa E., Zemojtel T., Buske O.J., Washington N.L. et al. Next-generation diagnostics and disease-gene discovery with the Exomiser. Nat. Protoc. 2015; 10:2004–2015. 26562621 10.1038/nprot.2015.124 PMC5467691

[16] Robinson P.N., Ravanmehr V., Jacobsen J.O.B., Danis D., Zhang X.A., Carmody L.C., Gargano M.A., Thaxton C.L., Biocuration Core UNC, Karlebach G. et al. Interpretable clinical genomics with a likelihood ratio paradigm. Am. J. Hum. Genet. 2020; 107:403–417. 32755546 10.1016/j.ajhg.2020.06.021 PMC7477017

[17] Amberger J.S., Bocchini C.A., Scott A.F., Hamosh A. OMIM.org: leveraging knowledge across phenotype-gene relationships. Nucleic Acids Res. 2019; 47:D1038–D1043. 30445645 10.1093/nar/gky1151 PMC6323937

[18] Bragin E., Chatzimichali E.A., Wright C.F., Hurles M.E., Firth H.V., Bevan A.P., Swaminathan G.J. DECIPHER: database for the interpretation of phenotype-linked plausibly pathogenic sequence and copy-number variation. Nucleic Acids Res. 2014; 42:D993–D1000. 24150940 10.1093/nar/gkt937 PMC3965078

[19] Köhler S., Carmody L., Vasilevsky N., Jacobsen J.O.B., Danis D., Gourdine J.-P., Gargano M., Harris N.L., Matentzoglu N., McMurry J.A. et al. Expansion of the Human Phenotype Ontology (HPO) knowledge base and resources. Nucleic Acids Res. 2019; 47:D1018–D1027. 30476213 10.1093/nar/gky1105 PMC6324074

[20] Fisher R.S., van Emde Boas W., Blume W., Elger C., Genton P., Lee P., Engel J. Epileptic seizures and epilepsy: definitions proposed by the International League Against Epilepsy (ILAE) and the International Bureau for Epilepsy (IBE). Epilepsia. 2005; 46:470–472. 15816939 10.1111/j.0013-9580.2005.66104.x

[21] Scheffer I.E., Berkovic S., Capovilla G., Connolly M.B., French J., Guilhoto L., Hirsch E., Jain S., Mathern G.W., Moshé S.L. et al. ILAE classification of the epilepsies: position paper of the ILAE Commission for Classification and Terminology. Epilepsia. 2017; 58:512–521. 28276062 10.1111/epi.13709 PMC5386840

[22] Helbig I., Lopez-Hernandez T., Shor O., Galer P., Ganesan S., Pendziwiat M., Rademacher A., Ellis C.A., Hümpfer N., Schwarz N. et al. A recurrent missense variant in AP2M1 impairs Clathrin-Mediated endocytosis and causes developmental and epileptic encephalopathy. Am. J. Hum. Genet. 2019; 104:1060–1072. 31104773 10.1016/j.ajhg.2019.04.001 PMC6556875

[23] Ganesan S., Galer P.D., Helbig K.L., McKeown S.E., O’Brien M., Gonzalez A.K., Felmeister A.S., Khankhanian P., Ellis C.A., Helbig I. A longitudinal footprint of genetic epilepsies using automated electronic medical record interpretation. Genet. Med. 2020; doi:10.1038/s41436-020-0923-1. 10.1038/s41436-020-0923-1 PMC7708303 32773773

[24] Galer P.D., Ganesan S., Lewis-Smith D., McKeown S.E., Pendziwiat M., Helbig K.L., Ellis C.A., Rademacher A., Smith L., Poduri A. et al. Semantic similarity analysis reveals robust gene-disease relationships in developmental and epileptic encephalopathies. Am. J. Hum. Genet. 2020; 107:683–697. 32853554 10.1016/j.ajhg.2020.08.003 PMC7536581

[25] Fisher R.S., Cross J.H., French J.A., Higurashi N., Hirsch E., Jansen F.E., Lagae L., Moshe S.L., Peltola J., Roulet Perez E. et al. Operational classification of seizure types by the International League Against Epilepsy: Position Paper of the ILAE Commission for Classification and Terminology. Epilepsia. 2017; 58:522–530. 28276060 10.1111/epi.13670

[26] Tudorache T., Nyulas C., Noy N.F., Musen M.A. WebProtégé: a collaborative ontology editor and knowledge acquisition tool for the web. Semantic web. 2013; 4:89–99. 23807872 10.3233/SW-2012-0057 PMC3691821

[27] Trinka E., Cock H., Hesdorffer D., Rossetti A.O., Scheffer I.E., Shinnar S., Shorvon S., Lowenstein D.H. A definition and classification of status epilepticus–Report of the ILAE Task Force on Classification of Status Epilepticus. Epilepsia. 2015; 56:1515–1523. 26336950 10.1111/epi.13121

[28] Engel J. Jr International League Against Epilepsy A proposed diagnostic scheme for people with epileptic seizures and with epilepsy: report of the ILAE Task Force on Classification and Terminology. Epilepsia. 2001; 42:796–803. 11422340 10.1046/j.1528-1157.2001.10401.x

[29] Pressler R.M., Cilio M.R., Mizrahi E.M., Moshé S.L., Nunes M.L., Plouin P., Vanhatalo S., Yozawitz E., Zuberi S.M. The ILAE classification of seizures & the epilepsies: modification for Seizures in the Neonate. 2019; Proposal from the ILAE Task Force on Neonatal Seizures. 10.1111/epi.16815 33522601

[30] Luders H., Acharya J., Baumgartner C., Benbadis S., Bleasel A., Burgess R., Dinner D.S., Ebner A., Foldvary N., Geller E. et al. Semiological seizure classification. Epilepsia. 1998; 39:1006–1013. 9738682 10.1111/j.1528-1157.1998.tb01452.x

[31] Nelson K.B., Ellenberg J.H. Predictors of epilepsy in children who have experienced febrile seizures. N. Engl. J. Med. 1976; 295:1029–1033. 972656 10.1056/NEJM197611042951901

[32] Uemura N., Okumura A., Negoro T., Watanabe K. Clinical features of benign convulsions with mild gastroenteritis. Brain Dev. 2002; 24:745–749. 12453597 10.1016/s0387-7604(02)00097-9

[33] Steering Committee on Quality Improvement and Management, Subcommittee on Febrile Seizures Febrile seizures: clinical practice guideline for the long-term management of the child with simple febrile seizures. Pediatrics. 2008; 121:1281–1286. 18519501 10.1542/peds.2008-0939

[34] Scheffer I.E., Berkovic S.F. Generalized epilepsy with febrile seizures plus. A genetic disorder with heterogeneous clinical phenotypes. Brain. 1997; 120:479–490. 9126059 10.1093/brain/120.3.479

[35] Seidel M.G., Kindle G., Gathmann B., Quinti I., Buckland M., van Montfrans J., Scheible R., Rusch S., Gasteiger L.M., Grimbacher B. et al. The European Society for Immunodeficiencies (ESID) registry working definitions for the clinical diagnosis of inborn errors of immunity. J. Allergy Clin. Immunol. Pract. 2019; 7:1763–1770. 30776527 10.1016/j.jaip.2019.02.004

[36] Tangye S.G., Al-Herz W., Bousfiha A., Chatila T., Cunningham-Rundles C., Etzioni A., Franco J.L., Holland S.M., Klein C., Morio T. et al. Human inborn errors of immunity: 2019 update on the classification from the international union of immunological societies expert committee. J. Clin. Immunol. 2020; 40:24–64. 31953710 10.1007/s10875-019-00737-x PMC7082301

[37] Arbabi A., Adams D.R., Fidler S., Brudno M. Identifying clinical terms in medical text using ontology-guided machine learning. JMIR Med. Inform. 2019; 7:e12596. 31094361 10.2196/12596 PMC6533869

[38] Haendel M.A., Balhoff J.P., Bastian F.B., Blackburn D.C., Blake J.A., Bradford Y., Comte A., Dahdul W.M., Dececchi T.A., Druzinsky R.E. et al. Unification of multi-species vertebrate anatomy ontologies for comparative biology in Uberon. J. Biomed. Semantics. 2014; 5:21. 25009735 10.1186/2041-1480-5-21 PMC4089931

[39] Ong E., Wang L.L., Schaub J., O’Toole J.F., Steck B., Rosenberg A.Z., Dowd F., Hansen J., Barisoni L., Jain S. et al. Modeling kidney disease using ontology: Perspectives from the KPMP. Nat. Rev. Nephrol. 2020; 16:686–696. 32939051 10.1038/s41581-020-00335-w PMC8012202

[40] Griese M., Irnstetter A., Hengst M., Burmester H., Nagel F., Ripper J., Feilcke M., Pawlita I., Gothe F., Kappler M. et al. Categorizing diffuse parenchymal lung disease in children. Orphanet J. Rare. Dis. 2015; 10:122. 26408013 10.1186/s13023-015-0339-1 PMC4582630

[41] Griese M., Seidl E., Hengst M., Reu S., Rock H., Anthony G., Kiper N., Emiralioğlu N., Snijders D., Goldbeck L. et al. International management platform for children's interstitial lung disease (chILD-EU). Thorax. 2018; 73:231–239. 29056600 10.1136/thoraxjnl-2017-210519

[42] Giannopoulou E., Katsila T., Mitropoulou C., Tsermpini E.-E., Patrinos G.P. Integrating next-generation sequencing in the clinical pharmacogenomics workflow. Front. Pharmacol. 2019; 10:384. 31024324 10.3389/fphar.2019.00384 PMC6460422

[43] Lloyd-Puryear M., Brower A., Berry S.A., Brosco J.P., Bowdish B., Watson M.S. Foundation of the newborn screening translational research network and its tools for research. Genet. Med. 2019; 21:1271–1279. 30393376 10.1038/s41436-018-0334-8

[44] Osumi-Sutherland D., Courtot M., Balhoff J.P., Mungall C. Dead simple OWL design patterns. J. Biomed. Semantics. 2017; 8:18. 28583177 10.1186/s13326-017-0126-0 PMC5460348

[45] Matentzoglu N., Balhoff J.P., Bello S.M., Boerkoel C.F., Bradford Y.M., Carmody L.C., Cooper L.D., Grove C.A., Harris N.L., Köhler S. et al. Phenotype Ontologies Traversing All The Organisms (POTATO) workshop aims to reconcile logical definitions across species. Zenodo. 2018; 10.5281/zenodo.2382757.

[46] Matentzoglu N., Balhoff J.P., Bello S.M., Bradford Y.M., Carmody L.C., Cooper L.D., Courtier-Orgogozo V., Cuzick A., Dahdul W.M., Diehl A.D. et al. Phenotype Ontologies Traversing All The Organisms (POTATO) workshop. 2019; 2nd edn.

[47] Gourdine J.-P.F., Brush M.H., Vasilevsky N.A., Shefchek K., Köhler S., Matentzoglu N., Munoz-Torres M.C., McMurry J.A., Zhang X.A., Robinson P.N. et al. Representing glycophenotypes: semantic unification of glycobiology resources for disease discovery. Database. 2019; 2019:baz114. 31735951 10.1093/database/baz114 PMC6859258

[48] Smith C.L., Eppig J.T. The Mammalian Phenotype Ontology as a unifying standard for experimental and high-throughput phenotyping data. Mamm. Genome. 2012; 23:653–668. 22961259 10.1007/s00335-012-9421-3 PMC3463787

[49] Osumi-Sutherland D., Marygold S.J., Millburn G.H., McQuilton P.A., Ponting L., Stefancsik R., Falls K., Brown N.H., Gkoutos G.V. The Drosophila phenotype ontology. J. Biomed. Semantics. 2013; 4:30. 24138933 10.1186/2041-1480-4-30 PMC3816596

[50] Schindelman G., Fernandes J.S., Bastiani C.A., Yook K., Sternberg P.W. Worm phenotype ontology: integrating phenotype data within and beyond the C. elegans community. BMC Bioinformatics. 2011; 12:32. 21261995 10.1186/1471-2105-12-32 PMC3039574

[51] Nenni M.J., Fisher M.E., James-Zorn C., Pells T.J., Ponferrada V., Chu S., Fortriede J.D., Burns K.A., Wang Y., Lotay V.S. et al. Xenbase: Facilitating the use of xenopus to model human disease. Front. Physiol. 2019; 10:154. 30863320 10.3389/fphys.2019.00154 PMC6399412

[52] Bradford Y., Conlin T., Dunn N., Fashena D., Frazer K., Howe D.G., Knight J., Mani P., Martin R., Moxon S.A.T. et al. ZFIN: enhancements and updates to the Zebrafish Model Organism Database. Nucleic Acids Res. 2011; 39:D822–D829. 21036866 10.1093/nar/gkq1077 PMC3013679

[53] Chang W.H., Mashouri P., Lozano A.X., Johnstone B., Husić M., Olry A., Maiella S., Balci T.B., Sawyer S.L., Robinson P.N. et al. Phenotate: crowdsourcing phenotype annotations as exercises in undergraduate classes. Genet. Med. 2020; 22:1391–1400. 32366968 10.1038/s41436-020-0812-7

[54] Robinson P.N., Haendel M.A. Ontologies, knowledge representation, and machine learning for translational research: recent contributions. Yearb Med. Inform. 2020; 29:159–162. 32823310 10.1055/s-0040-1701991 PMC7442528

[55] McDonald C.J., Huff S.M., Suico J.G., Hill G., Leavelle D., Aller R., Forrey A., Mercer K., DeMoor G., Hook J. et al. LOINC, a universal standard for identifying laboratory observations: a 5-year update. Clin. Chem. 2003; 49:624–633. 12651816 10.1373/49.4.624

[56] Zhang X.A., Yates A., Vasilevsky N., Gourdine J.P., Callahan T.J., Carmody L.C., Danis D., Joachimiak M.P., Ravanmehr V., Pfaff E.R. et al. Semantic integration of clinical laboratory tests from electronic health records for deep phenotyping and biomarker discovery. npj Digital Med. 2019; 2:32. 10.1038/s41746-019-0110-4 PMC6527418 31119199

[57] Mishra R., Burke A., Gitman B., Verma P., Engelstad M., Haendel M.A., Alevizos I., Gahl W.A., Collins M.T., Lee J.S. et al. Data-driven method to enhance craniofacial and oral phenotype vocabularies. J. Am. Dent. Assoc. 2019; 150:933–939. 31668172 10.1016/j.adaj.2019.05.029 PMC6827714

[58] Bastarache L., Hughey J.J., Goldstein J.A., Bastraache J.A., Das S., Zaki N.C., Zeng C., Tang L.A., Roden D.M., Denny J.C. Improving the phenotype risk score as a scalable approach to identifying patients with Mendelian disease. J. Am. Med. Inform. Assoc. 2019; 26:1437–1447. 31609419 10.1093/jamia/ocz179 PMC6857501

[59] Tang X., Chen W., Zeng Z., Ding K., Zhou Z. An ontology-based classification of Ebstein's anomaly and its implications in clinical adverse outcomes. Int. J. Cardiol. 2020; 316:79–86. 32348812 10.1016/j.ijcard.2020.04.073

[60] Kafkas Ş., Abdelhakim M., Hashish Y., Kulmanov M., Abdellatif M., Schofield P.N., Hoehndorf R. PathoPhenoDB, linking human pathogens to their phenotypes in support of infectious disease research. Sci. Data. 2019; 6:79. 31160594 10.1038/s41597-019-0090-x PMC6546783

[61] Son J.H., Xie G., Yuan C., Ena L., Li Z., Goldstein A., Huang L., Wang L., Shen F., Liu H. et al. Deep phenotyping on electronic health records facilitates genetic diagnosis by clinical exomes. Am. J. Hum. Genet. 2018; 103:58–73. 29961570 10.1016/j.ajhg.2018.05.010 PMC6035281

[62] Dhombres F., Bodenreider O. Interoperability between phenotypes in research and healthcare terminologies–Investigating partial mappings between HPO and SNOMED CT. J. Biomed. Semantics. 2016; 7:3. 26865946 10.1186/s13326-016-0047-3 PMC4748471

[63] Thompson R., Papakonstantinou Ntalis A., Beltran S., Töpf A., de Paula Estephan E., Polavarapu K., ’t Hoen P.A.C., Missier P., Lochmüller H. Increasing phenotypic annotation improves the diagnostic rate of exome sequencing in a rare neuromuscular disorder. Hum. Mutat. 2019; 40:1797–1812. 31231902 10.1002/humu.23792

[64] Murphy S.N., Weber G., Mendis M., Gainer V., Chueh H.C., Churchill S., Kohane I. Serving the enterprise and beyond with informatics for integrating biology and the bedside (i2b2). J. Am. Med. Inform. Assoc. 2010; 17:124–130. 20190053 10.1136/jamia.2009.000893 PMC3000779

[65] Voss E.A., Makadia R., Matcho A., Ma Q., Knoll C., Schuemie M., DeFalco F.J., Londhe A., Zhu V., Ryan P.B. Feasibility and utility of applications of the common data model to multiple, disparate observational health databases. J. Am. Med. Inform. Assoc. 2015; 22:553–564. 25670757 10.1093/jamia/ocu023 PMC4457111

[66] Callahan T.J., Wyrwa J.M., Vasilevsky N.A., Bennett T.D., Kahn M.G. 2020; OMOP2OBOaccessed 11 October 2020 https://zenodo.org/record/3902767.

[67] Shefchek K.A., Harris N.L., Gargano M., Matentzoglu N., Unni D., Brush M., Keith D., Conlin T., Vasilevsky N., Zhang X.A. et al. The Monarch Initiative in 2019: an integrative data and analytic platform connecting phenotypes to genotypes across species. Nucleic Acids Res. 2019; 48:D704–D715. 10.1093/nar/gkz997 PMC7056945 31701156

